# One-and-a-Half Syndrome in a Case of Brainstem Bleed

**DOI:** 10.7759/cureus.53193

**Published:** 2024-01-29

**Authors:** Balamurugan Nathan, Ajithkumar Rajendran, Ezhilkugan G

**Affiliations:** 1 Emergency Medicine, Jawaharlal Institute of Postgraduate Medical Education and Research, Puducherry, IND

**Keywords:** emergency medicine, gaze palsy, brainstem stroke, internuclear ophthalmoplegia, one-and-a-half syndrome

## Abstract

One-and-a-half syndrome (OHS) is a horizontal gaze palsy in one direction with internuclear ophthalmoplegia (INO) in the other. The only eye movement possible is the abduction of the contralateral eye with nystagmus. The usual structures affected are the medial longitudinal fasciculus and paramedian pontine reticular formation or the abducens nucleus. Most commonly, the OHS is caused by ischemia and demyelinating lesions. The other causes include infectious, neoplastic, and rarely traumatic. We report a case of a 42-year-old non-compliant hypertensive female who presented with giddiness, projectile vomiting, and right-sided hemiparesis and was found to have OHS on cranial nerve examination in the emergency department (ED). In the ED, the presence of complete horizontal gaze palsy in one direction with INO in the other direction should raise suspicion of a brainstem pathology.

## Introduction

Fisher first described the one-and-a-half syndrome (OHS) in the year 1967 [1]. OHS is characterized by the presence of horizontal gaze palsy in the eye in one direction with internuclear ophthalmoplegia (INO) in the other direction. The structures affected in OHS are the paramedian pontine reticular formation (PPRF) or the abducens nucleus and the medial longitudinal fasciculus [2]. The only movement possible is the abduction of the other eye with nystagmus. Recognizing this syndrome in the emergency department (ED) allows for early diagnosis and will help to localize the lesion to the brainstem.

## Case presentation

**Figure 1:**
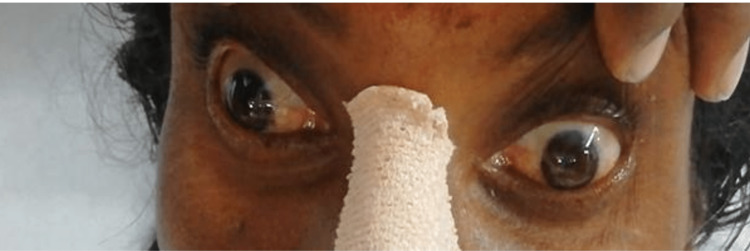
Left eye abduction palsy when the patient was asked to look toward the left Note the adduction palsy in the right eye.

**Figure 2:**
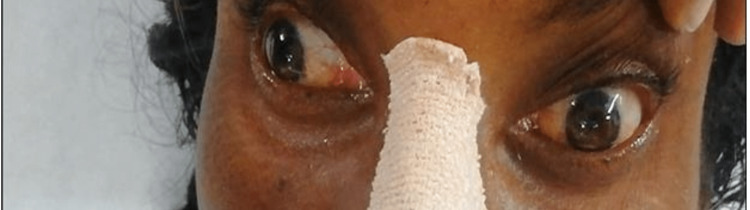
On right gaze, impaired adduction of the left eye with nystagmus of the abducting right eye

A 42-year-old female who was recently diagnosed hypertensive and not on medications presented to the ED with an inability to use the right upper and lower limbs for four hours. She had associated giddiness, projectile vomiting, and numbness of the entire right half of the body. On general physical examination, she was conscious and oriented, with a pulse rate of 90 beats per minute and a blood pressure of 170/110 mmHg. Central nervous system examination revealed bilaterally equal and reacting pupils. She had complete horizontal gaze palsy involving the left eye and nystagmus of the right eye on abduction (Figures 1, 2). She was also noted to have right upward gaze palsy.

**Figure 3:**
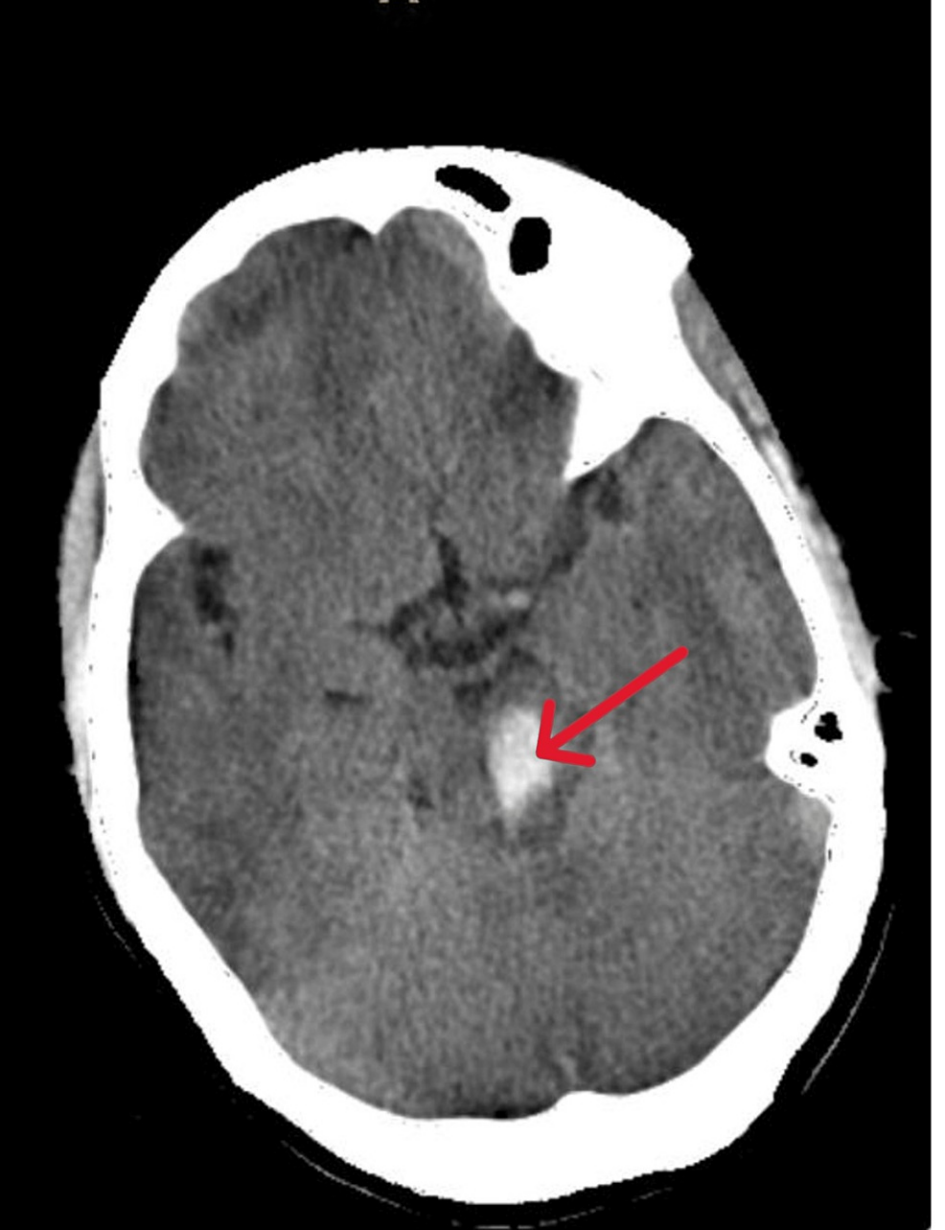
Non-contrast CT brain showing bleed in the mid-brain region Arrow points to the bleed.

**Figure 4:**
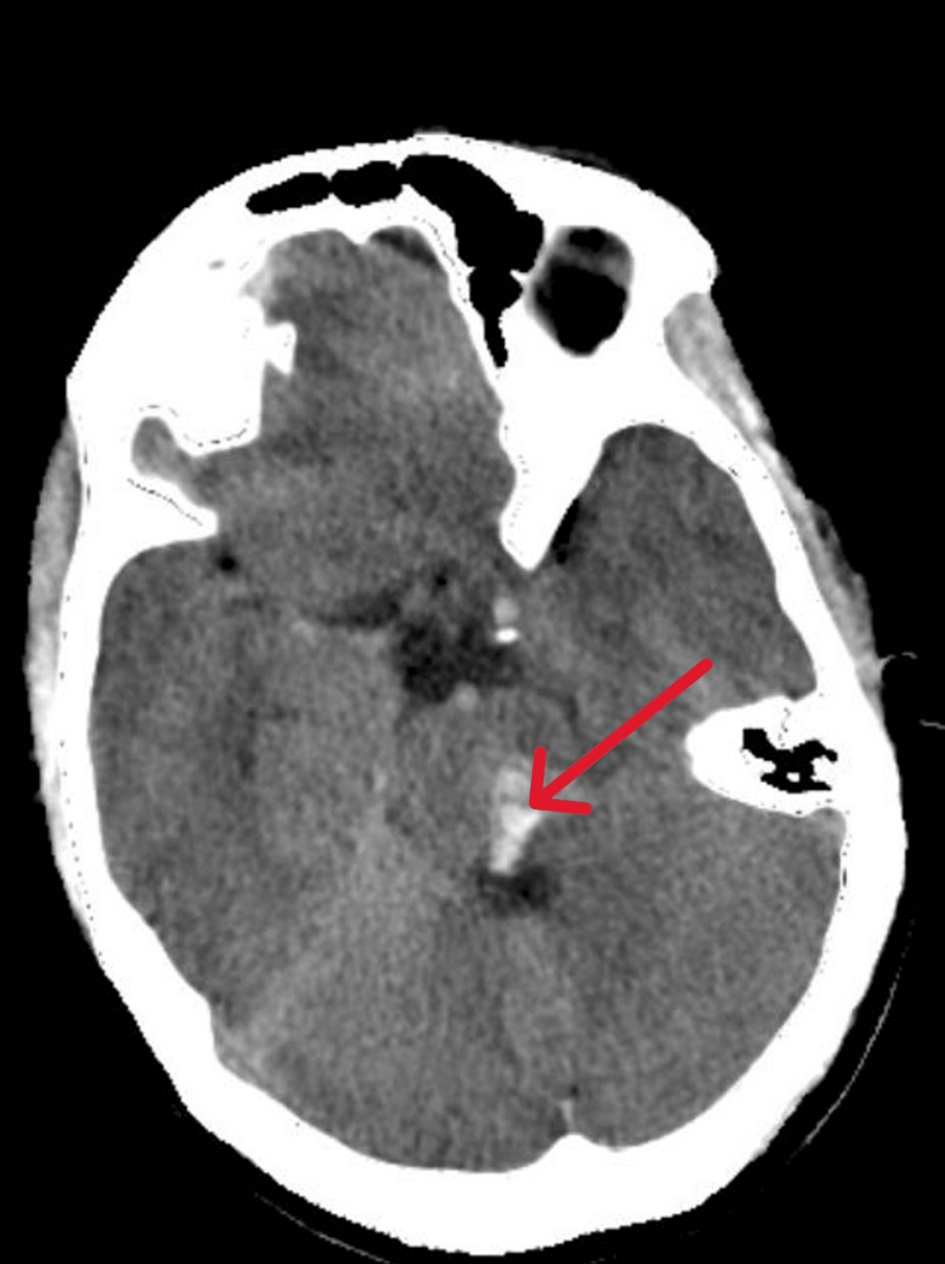
Non-contrast CT brain showing bleed in the pons Arrow points to the bleed.

From Figures 1, 2, it is evident that the only ocular movement possible in our patient was the abduction of the right eye. On motor examination, right upper limb power was 3/5 with a distal handgrip of 70%, and right lower limb power was 2/5 with preserved power on the left side. The plantar response was mute bilaterally. The sensory examination was normal. Based on these neurological findings, she was considered to have an acute onset of right-sided hemiparesis with OHS, probably due to brainstem stroke. Non-contrast CT brain revealed hemorrhage in the left midbrain and upper pons (Figures 3, 4).

She was managed with antihypertensives, and she was discharged after a month of hospital stay. Her ocular movements also have improved at the two-and-a-half-year follow-up (telephonic assessment), and she is able to do her routine activities.

## Discussion

To understand OHS, one needs to understand the following structures and their function.

Abducens nuclei

This is located in the pons and is responsible for the innervation of the lateral rectus muscle. The medial longitudinal fasciculus that innervates the contralateral eye also originates from the abducens nuclei.

PPRF

PPRF is the supranuclear center situated close to the ipsilateral abducens nucleus and the ventral MLF that gets the impulses from the visual area of the parietal and frontal lobes and controls horizontal conjugate eye movements [3].

Medial longitudinal fasciculus

This tract, as described above, originates from the abducens nucleus and crosses over the midline to innervate the contralateral medial rectus. 

**Figure 5:**
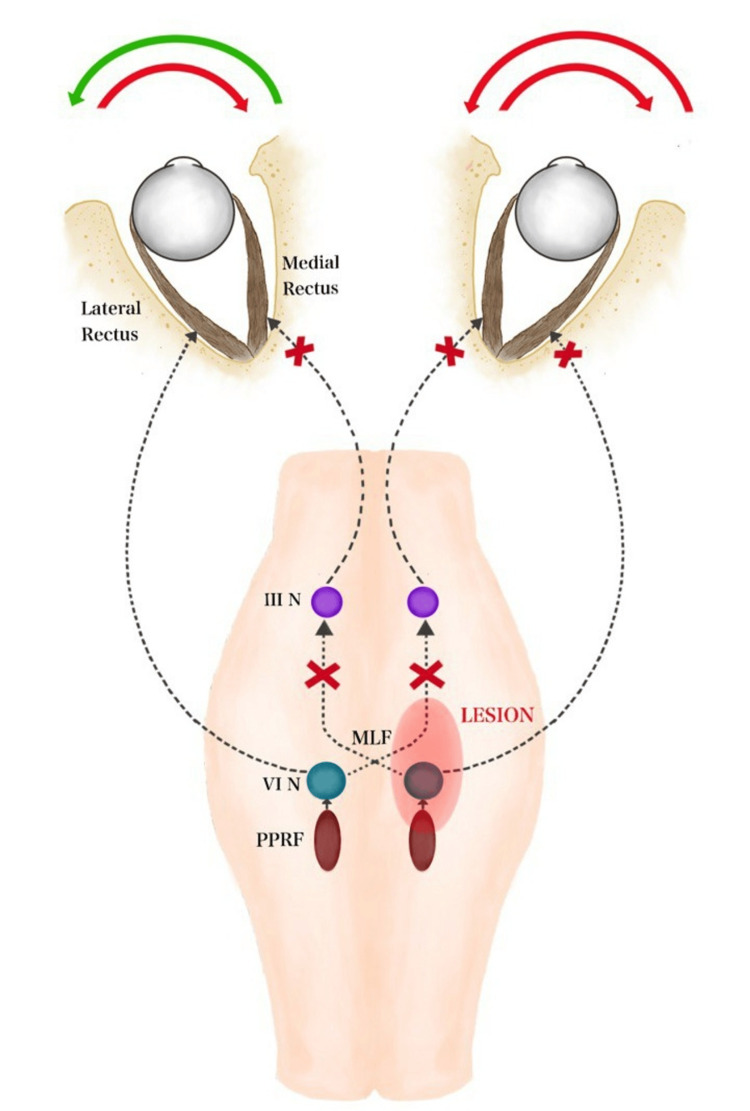
Schematic diagram showing the ocular pathways involved in one-and-half syndrome The brown oval represents the PPRF. The cyan blue/gray circle represents the sixth nerve nucleus. The purple circle represents the third nerve nucleus. The dotted line represents the medial longitudinal fasciculus. N, nucleus; PPRF, paramedian pontine reticular formation (Image credit: redrawn from reference [3] by Dr. Swetha Ramesh)

When the lesion involves PPRF or an abducens nucleus on one side, this results in impaired abduction of the ipsilateral eye. Since the lesion also affects the ipsilateral medial longitudinal fasciculus that has crossed over from the contralateral abducens nucleus, this results in weakness of adduction in the ipsilateral eye and nystagmus in the abducting eye. This results in complete horizontal gaze palsy of the ipsilateral eye (one). Since the lesion also involves the abducens on the same side, the medial longitudinal fasciculus that originates from the ipsilateral abducens nucleus that supplies the contralateral eye is affected. Hence, the adduction of the contralateral eye is affected (half movement of the other eye). So, the only movement possible is the abduction of the contralateral eye (Figure 5).

OHS was first coined by Fischer in 1967. The isolated form of OHS is uncommon, and this syndrome is usually accompanied by cranial nerve palsies, hemiplegia, or hemihypesthesia [1]. Brainstem lesions affecting horizontal eye movements can be classified into three groups: (a) lateral gaze palsy, (b) INO, and (c) OHS [4].

The common causes of OHS are ischemia and demyelinating lesions. Other causes of the disease are neurogenic or metastatic tumors, encephalitis, basilar meningitis, Wernicke encephalopathy, progressive supranuclear palsy, radiotherapy, syringobulbia and trauma, cavernoma, racemose neurocysticercosis, and tuberculoma [5-7]. Pseudo OHS is also reported in cases of myasthenia gravis, where patients present with the features of INO with ptosis [8]. Levator palpebrae, medial recti, and superior recti are thought to be the most commonly affected extraocular muscles in myasthenia gravis. Hyperleukocytosis resulting in brain infarction and thus presenting as OHS is also reported [9]. OHS also has been reported to be the first manifestation of systemic lupus erythematosus (SLE) [10].

OHS is of three types, and they are as follows: type 1 is conjugate horizontal gaze palsy (CHGP) plus INO, type 2 is CHGP and spared adduction of one eye or pupillary disturbances, and type 3 is CHGP plus unilateral vertical paresis or other combinations [11]. A lesion involving the PPRF, MLF, and its ipsilateral facial nerve fascicle around the area of the facial colliculus is reported as the eight-and-a-half syndrome [12]. OHS plus facial nerve palsy with contralateral hemiparesis called the nine syndrome was observed in a case due to lacunar pontine infarction [13]. Eight-and-a-half syndrome plus the ipsilateral fifth cranial nerve (trigeminal nerve) involvement called thirteen-and-a-half syndrome due to lymphoma has been reported [14]. OHS plus involvement of bilateral seventh cranial nerve called fifteen-and-a-half syndrome caused by bilateral pontine tegmental lesion is also reported [15]. OHS plus one-sided seventh cranial nerve, hemiparesis, and one-sided hearing loss form sixteen-and-a-half syndrome. This was observed in a case of metastatic pons tumor [16]. OHS plus bilateral facial nerve palsy with one-sided fifth nerve palsy due to lacunar infarcts are also reported in the literature [17]. According to Wall and Wray, contralateral hemiparesis is found in 30% of cases with OHS, while hemisensory loss is in 35% [2]. Patients with OHS caused by multiple sclerosis, cerebrovascular disorders, and brainstem lacunar infarct have a good outcome; the majority of them recover without sequelae [3]. MRI with angiogram is the modality of choice for the diagnosis. The disabling diplopia can be managed conservatively by temporary monocular occlusion until the diplopia resolves by itself [18]. OHS due to pontine bleed has been reported to show complete recovery in six months [12]. In our scenario also, the patient’s ocular deficit has improved, and she is carrying out her routine activities.

## Conclusions

In the ED, recognition of the extraocular muscle abnormality might give a clue to the underlying structure involved and thus localize the lesion. The presence of complete gaze palsy in one direction with INO in the other direction should raise the suspicion of brainstem involvement, especially pons. OHS, due to bleed, has a good prognosis.
